# The tumor suppressor CREBBP and the oncogene *MYCN* cooperate to induce malignant brain tumors in mice

**DOI:** 10.1038/s41389-023-00481-3

**Published:** 2023-07-05

**Authors:** Melanie Schoof, Gefion Dorothea Epplen, Carolin Walter, Annika Ballast, Dörthe Holdhof, Carolin Göbel, Sina Neyazi, Julian Varghese, Thomas Karl Albert, Kornelius Kerl, Ulrich Schüller

**Affiliations:** 1 Research Institute Children`s Cancer Center, Hamburg, Germany; 2 Department of Pediatric Hematology and Oncology, University Medical Center Hamburg-Eppendorf, Hamburg, Germany; 3 Institute of Medical Informatics, University of Münster, Münster, Germany; 4 Department of Pediatric Hematology and Oncology, University Children’s Hospital Münster, Münster, Germany; 5 Institute of Neuropathology, University Medical Center Hamburg-Eppendorf, Hamburg, Germany

**Keywords:** Pattern formation, Cancer models

## Abstract

The tumor suppressor and chromatin modifier cAMP response element-binding protein binding protein (CREBBP) and v-myc avian myelocytomatosis viral oncogene neuroblastoma derived homolog (MYCN), a member of the MYC oncogene family, are critically involved in brain development. Both genes are frequently mutated in the same tumor entities, including high-grade glioma and medulloblastoma. Therefore, we hypothesized that alterations in both genes cooperate to induce brain tumor formation. For further investigation, *hGFAP-cre::Crebbp*^*Fl/Fl*^*::lsl-MYCN* mice were generated, which combine *Crebbp* deletion with overexpression of *MYCN* in neural stem cells (NSCs). Within eight months, these animals developed aggressive forebrain tumors. The first tumors were detectable in the olfactory bulbs of seven-day-old mice. This location raises the possibility that presumptive founder cells are derived from the ventricular-subventricular zone (V-SVZ). To examine the cellular biology of these tumors, single-cell RNA sequencing was performed, which revealed high intratumoral heterogeneity. Data comparison with reference CNS cell types indicated the highest similarity of tumor cells with transit-amplifying NSCs or activated NSCs of the V-SVZ. Consequently, we analyzed V-SVZ NSCs of our mouse model aiming to confirm that the tumors originate from this stem cell niche. Mutant V-SVZ NSCs showed significantly increased cell viability and proliferation as well as reduced glial and neural differentiation in vitro compared to control cells. In summary, we demonstrate the oncogenic potential of a combined loss of function of CREBBP and overexpression of *MYCN* in this cell population. *hGFAP-cre::Crebbp*^*Fl/Fl*^*::lsl-MYCN* mice thus provide a valuable tool to study tumor-driving mechanisms in a key neural stem/ progenitor cell niche.

## Introduction

Alterations of cAMP response element-binding protein binding protein (*CREBBP*) and v-myc avian myelocytomatosis viral oncogene neuroblastoma derived homolog (*MYCN*) have been identified in a large variety of human tumors in numerous cancer genome studies (COSMIC - Catalogue Of Somatic Mutations In Cancer (RRID:SCR_002260), ref. [1]; ICGC Data Portal (RRID:SCR_021722), ref. [2]). Both genes have been described to be altered in the same brain tumor entities, but only rarely in the same tumors. Entities with recurrent alterations of *MYCN* and *CREBBP* are for example medulloblastoma and malignant glioma [3–5]. Additionally, both genes are known to be essential in brain development [5–8].

CREBBP and its closely related paralog p300 are ubiquitously expressed lysine acetyltransferases. They are involved in the regulation of fundamental cellular functions, both as chromatin modifiers and transcriptional coactivators and via non-transcriptional effects [9]. CREBBP facilitates transcription through histone acetylation and serves as a scaffold bridging the basal transcriptional machinery with a multitude of different transcription factors. CREBBP has thereby global effects on transcriptional regulation and the epigenetic landscape of cells, which might explain the involvement of CREBBP in tumor development. The specific roles of CREBBP in cancer development have only partly been defined, including pro-oncogenic as well as tumor-suppressive functions [5, 9]. Mutations in genes involved in histone acetylation – including *CREBBP* – are overrepresented in Sonic hedgehog (SHH)-activated medulloblastoma [4, 5]. Epigenetic dysregulation in general is frequently observed in many forms of childhood cancer. Somatic mutations driving pediatric cancers are enriched in genes encoding the epigenetic machinery [10]. Germline mutations in these genes have been associated with a growing number of neurodevelopmental disorders [7]. Heterozygous loss of *CREBBP* in the germline is the cause of Rubinstein-Taybi syndrome, and various benign and malignant brain tumors including glioma and medulloblastoma have been reported in those patients [11, 12].

The protein MYCN belongs to the family of MYC transcription factors, which function as transcriptional master regulators in a variety of cellular processes [6]. Enhanced and deregulated expression of *MYCN*, often resulting from gene amplification, drives the development of multiple cancers, including tumors of the nervous system, both in children and adults [6, 13–16]. The 2021 WHO classification of tumors of the central nervous system (CNS) lists *MYCN* among the genes characteristically altered in diffuse pediatric-type glioma, spinal ependymoma, as well as SHH-activated and non-WNT/non-SHH medulloblastoma [17]. Recently, an important role of MYCN was also described for embryonal tumors with multi-layered rosettes (ETMR) [18].

Previous mouse models, which carry a knockout of Crebbp or an overexpression of MYCN in specific neural cell populations, do not develop brain tumors [8, 19]. However, alterations of both genes have been described in the same tumor entities [3–5]. Therefore, we decided to study the effects of *MYCN* overexpression and a simultaneous loss of CREBBP to understand tumor driving mechanisms.

To study the combined effects of *Crebbp* and *MYCN* alterations, we established and characterized a novel mouse model, *hGFAP-cre::Crebbp*^*Fl/Fl*^*::lsl-MYCN*. In these mice, biallelic loss of *Crebbp* and overexpression of human *MYCN* is targeted to a wide range of neural stem/progenitor cell populations from embryonic day 13.5 onwards, to their progeny, including neurons, glia, and mature astrocytes [20]. With short postnatal latency, the animals developed malignant brain tumors arising from the olfactory bulbs (OBs). This location raises the possibility that presumptive founder cells are derived from the ventricular-subventricular zone (V-SVZ), the largest germinal region in the adult rodent brain or its embryonal antecedents [21–23]. V-SVZ neural stem cells (V-SVZ NSCs) give rise to different types of OB interneurons throughout life, reflecting their regional identity determined by distinct embryonic origins in the pallium, ganglionic eminences, and septum [21]. Adult quiescent NSCs (qNSCs) become activated NSCs (aNSCs) and—via rapidly dividing transit amplifying cells (TACs)—generate neuroblasts, which migrate along the rostral migratory stream (RMS) to the OB, where they differentiate into OB interneurons [21, 24, 25]. Recent single-cell RNA sequencing data suggest that qNSCs arise from embryonal progenitors showing transcriptional similarity with adult aNSCs [26]. Tumor cells from our model closely resemble adult V-SVZ aNSCs / TACs when compared to adult V-SVZ/OB cells on the transcriptional level [24], indicating that these cell populations or their embryonal progenitors are candidate cells of origins of the OB tumors.

## Methods

### Transgenic animals

Both *hGFAP-cre*::*Crebbp*^*Fl/Fl*^ [8, 20, 27] and *hGFAP-cre::lsl-MYCN* [19, 20, 28] mouse lines have previously been described. These mice were crossed to generate *hGFAP-cre*::C*rebbp*^*Fl/Fl*^*::lsl-MYCN* mice. The genotype was confirmed by PCR using genomic DNA from tail or ear biopsies utilizing the following primers: Cre: TCCGGGCTGCCACGACCAA, GGCGCGGCAACACCATTTT, *Crebbp*: CCTCTGAAGGAGAAACAAGCA, ACCATCATTCATCAGTGGACT; *MYCN*: ACCACAAGGCCCTCAGTACC, TGGGACGCACAGTGATGG as previously described [8, 28]. Mice were maintained on a C57BL/6J background. Both male and female mice were examined. All experiments were performed according to applicable animal protection laws and were approved by the Government of Hamburg, Germany (Reference TVA N099/2019).

### Cell culture experiments

For primary culture of tumor cells, OB cells of 3- to 4-week-old *hGFAP-cre::Crebbp*^*Fl/Fl*^*::lsl-MYCN* mice were isolated and dissociated mechanically and enzymatically with Accutase (#A1110501, Thermo Fisher) for 10 min at RT. The cell suspension was filtered by a 40 µm cell strainer (#352340, Corning) and cells were seeded in a T25 flask (#83.3919.502, Sarstedt). They were cultured in serum-free medium with growth factors (NSC medium: DMEM/F12+Glutamax supplement (#31331093, Thermo Fisher), 2% B27 supplement (#17504001, Thermo Fisher), 4% HEPES buffer (#15630056, Life Technologies), 0.1% MEM non-essential amino acids (#M7145-100ML, Sigma), 0.1% penicillin/streptomycin (#15140122, Life Technologies), 0.02% epidermal growth factor (EGF, #AF-100-15, Peprotech) and fibroblast growth factor (FGF, #100-18c, Peprotech)) for up to 6 weeks. After freezing and thawing, the newly established tumor cell lines were dissociated by using Accutase and used for further experiments. For experiments with secondary NSCs, the V-SVZ of two to four 5-day-old *hGFAP-cre::Crebbp*^*Fl/Fl*^*::lsl-MYCN* mice and two to four *Crebbp*^*Fl/Fl*^*::lsl-MYCN* control littermates was dissociated and pooled within mutant and control group, respectively. In order to remove non-NSC cells, cells were cultured in NSC medium. After DNA isolation with a NucleoSpin Tissue Kit (#740952.50, Machery-Nagel), successful recombination in the V-SVZ cells was confirmed via PCR utilizing the following primers: *Crebbp* recombined: ACCATCATTCATCAGTGGAC, ATGTAAGAACAGCCCCAAAC; *MYCN* recombined: GCCCGCGGTGATGCCTTTGAGG, CGGGGACTGGGCGGTGGAAC as previously described [20, 28]. After 7 days in culture, the neurospheres were split into single cells using Accutase, and further experiments were performed.

### Histology and immunohistochemistry

For mouse brain analysis, brain tissue was prepared for staining according to standardized procedures by fixation in 4% formaldehyde (#70002-4-10, Grimm med. Logistik) (minimum 12 h), dehydration and paraffin embedding. Sections of 4 μm were cut according to standard laboratory protocols. For histological analysis of tumor cells, cell pellets were fixed in 4% paraformaldehyde (PFA) (#CP10.2, Roth) for 30 min, embedded in agarose (#840004, Biozym) and dehydrated. Sections of 4 μm were cut. Haematoxylin/eosin (H&E) staining followed a standard protocol. Immunohistochemical staining was performed on a staining machine (Ventana BenchMark XT System, Roche), or using a staining kit (Novolink Polymer Detection System, #RE7140-CE, Leica) according to manufacturer’s instructions. Primary antibodies included: Ki67 (#ab15580, Abcam, 1:100), MYCN (#51705, Cell Signaling, 1:100), CREBBP (C-1) (#sc-7300, Santa Cruz, 1:50), SOX2 (#ab97959, Abcam, 1:100), OLIG2 (#ab9610, Millipore, 1:200), MAP2 (#M4403, Sigma, 1:3000), GFAP (#M0761, Dako, 1:200), S100A1 (#Z0311, Dako, 1:100), OTX2 (Thermo Scientific, #1H12C4B5, 1:2000). Chromogenic detection was performed by secondary antibodies and 3,3′-Diaminobenzidine (DAB). Stained slides were analyzed on a microscope (BX43F, Olympus), photographed (OLYMPUS cellSens Entry 1.15 software) and measured using ImageJ.

### Immunofluorescence staining

For immunofluorescence staining cells were blocked in 10% normal goat serum (NGS) (#S26-100ML, Merck Millipore) and visualized using the following primary antibodies: anti-GFAP (#14-9892, Invitrogen, mouse IgG1, 1:500), anti-TUBB3 (#T2200, Sigma, rabbit IgG, 1:200), anti-Ki67 (#ab16667, Abcam, rabbit, 1:200). Fluorescent detection was achieved by species-specific fluorophore-linked secondary antibodies: Alexa 555 α-mouse (#CST4409, Cell Signaling, 1:500 and Alexa fluor 488 α-rabbit (#CST4412, Cell Signaling, 1:500). DAPI (#28718–90-3, Roth, 1:1000) was used to stain cell nuclei. A microscope (Eclipse Ti2-E, Nikon) was used for analyses.

### Viability assay

To assess cell viability, cells were seeded in a density of 5 × 10^4^ cells in 100 μl NSC medium per well in a 96-well plate (#136101, Thermo Scientific). A CellTiter-Glo® Luminescent Cell Viability Assay (#G7571, Promega) was conducted according to the manufacturer’s instructions. The assay is read by luminescence, which is proportional to the amount of ATP present. The latter is proportional to the count of metabolically active cells. Luminescence was measured at 0, 24, 48, and 72 h post-seeding using a microplate reader (infinite M200, Tecan).

### In vitro differentiation assay

To facilitate cell adhesion, coverslips were coated with poly-L-ornithine (#P3655-100MG, Sigma) overnight in a freezer, washed with PBS and coated with laminin (#L2020-1MG, Sigma Aldrich) for 1 h at 37 °C beforehand. Subsequently, cells were cultured in medium containing 10% fetal calf serum (FCS) (#10270106, Life Technol.) to induce differentiation. After 7 days in culture, cells were fixed by PFA (10 min). Immunofluorescence staining was performed, and 10 representative pictures of every coverslip were taken and analyzed using NIS-Elements AR software (5.11.03).

### Tumor cell transplantation

For tumor cell injection, tumor cells were dissociated with Accutase, washed and resuspended in a solution of NSC Medium and Matrigel (#356234, Corning). Under anesthesia, the skull bone of recipient mice (8-week-old, CD1 nu/nu mice) was punctured and 1 × 10^6^ cells were stereotactically injected as previously described [29]. Remaining tumor cells were used as a growth control to confirm that cells survived the procedure. Animals were observed for signs of tumor development and sacrificed after 8 months. For details of mouse brain analysis see histology and immunohistochemistry.

### Single-cell RNA sequencing and data analysis

OB tumors of a 90-day-old male and a 100-day-old female *hGFAP-cre::Crebbp*^*Fl/Fl*^*::lsl-MYCN* mouse were isolated and minced with scalpels on ice. For enzymatic dissociation, 5 ml Papain (#LK003178, Worthington-Biochem) in DMEM/F12 (#21331020, Life Technologies) solution, supplemented with 1000 U DNase (#10104159001, Roche), was added to each sample, followed by incubation at 37 °C in 5% CO2 for 30 min. The cell solution was then passed through a 40 µm cell strainer. Red blood cells were lysed with ACK lysis buffer (#A1049201, Thermo Fisher) (5 min at 4 °C). The resulting single-cell suspensions were stained with 7-aminoactinomycin D (7-AAD) (#699350, Invitrogen). Non-viable, 7-AAD-positive cells were removed by fluorescence-activated cell sorting. Approximately 10,000 vital cells were used as input for scRNA-seq. Single-index libraries were generated with Chromium Single Cell 3′ v3.1 technology (10x Genomics) and sequenced using a NextSeq 2000 sequencing instrument (high-throughput kit, 100 cycles) at the Genomics Core Facility (University Hospital Münster, Germany) after quality control using a Tapestation 2200 (Agilent Technologies). The samples were analyzed with the 10x Genomics CellRanger v6.0.2 pipeline [30] and Seurat R package v4.0.5 (ref. [31]). Raw data were converted to fastq format with the CellRanger mkfastq function and then aligned against the murine reference transcriptome mm10 v2020-A with CellRanger count and default values. Seurat objects were generated for both samples based on the following filter criteria: at least three cells, a minimum feature count of 200, and cells with < 25% of mitochondrial genes. Outlier cells with a high nCount_RNA value were classified as doublets and removed (threshold: 30,000-35,000). The filtered data were then normalized, integrated, and clustered with Seurat, using a resolution parameter of 0.5. Feature plots, UMAPs and heatmap visualizations were created with Seurat functions; a cluster-based cell type annotation was conducted based on the expression of characteristic marker genes per cell type. Finally, a logistic regression analysis based on the approach of Young et al. [32] was used to calculate similarity scores between the original murine clusters and a reference dataset by Mizrak et al. [24].

### Comparison of human ONBs and mouse tumors

Global DNA methylation data of mouse and human tumors generated by Illumina array sequencing (450k or mouse methylation bead chip array, respectively) was used for creating copy number variation (CNV) profiles. Human data originates from Capper and Engel et al. [33]. CNV data was generated with the conumee package in R.

Mouse methylation profiles were generated with the in-house established pipeline for human material and the mouse methylation bead chip array MM285 (Illumina) was employed. Mouse CNV profiles were generated similar to human CNVs with an in-house adapted code and a custom reference set.

### Statistical analysis

Statistical analysis was conducted with GraphPad Prism software (RRID:SCR_002798). All observations were made in at least three independent animals or experiments. For cell culture experiments and animal studies, all analyses were permored in a blinded fashion without knowlodge of the genotype. No further randomization was needed. If not stated otherwise, all data presented are mean ± SD, with *n* = 3 for each group. Each data point represents an individual animal or an independent experiment. If not stated otherwise, the unpaired *t* test (two-tailed) was applied to compare the means of two groups. *P*-values < 0.05 were considered significant (*p < 0.05; **p < 0.01; ***p < 0.001; ****p < 0.0001).

## Results

### Functional loss of CREBBP and MYCN overexpression induce early onset brain tumors in mice

**Fig. 1:**
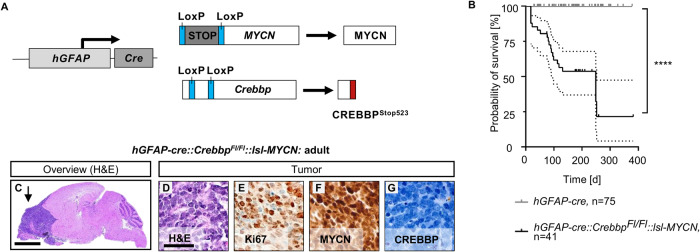
Overexpression of *MYCN* and deletion of CREBBP via *hGFAP-cre* induces malignant brain tumors in mice. **A** Schematic drawing of *hGFAP-cre::Crebbp*^*Fl/Fl*^*::lsl-MYCN* mice. **B** Kaplan–Meyer survival curves for the transgenic mouse model. *hGFAP-cre* (*n* = 75) vs *hGFAP-cre::Crebbp*^*Fl/Fl*^*::lsl-MYCN* (*n* = 41). ****p < 0.0001, log-rank test. Area between dotted line: 95% CI. **C** Representative histological overview image of an adult *hGFAP-cre::Crebbp*^*Fl/Fl*^*::lsl-MYCN* mouse brain. H&E, haematoxylin and eosin. Arrow marks the tumor. **D**–**G** High power images and immunohistochemistry (IHC: Ki67 (**E**), MYCN (**F**), CREBBP (**G**)) of the forebrain tumor. Scale bar corresponds to 2 mm (overview) and 50 μm (high power images).

We generated a conditional knockout mouse model (*hGFAP-cre::Crebbp*^*Fl/Fl*^*::lsl-MYCN)* that combines homozygous deletion of *Crebbp* and biallelic expression of exogenous human *MYCN* using a human *GFAP* promoter-controlled cre transgenic line (*hGFAP-cre*) to drive recombination [20]. The *hGFAP* promoter is expressed in neural precursor cells from embryonic day 13.5 onwards and remains active in mature astrocytes. In these cells, cre recombinase (cre) induces recombination between the loxP sites surrounding exon 7 in the *Crebbp* gene and a STOP codon upstream of the inserted human *MYCN* gene. Translation thus leads to a severely shortened, non-functional CREBBP protein and at the same time causes the expression of exogenous human MYCN (Fig. 1A).

Survival of *hGFAP-cre::Crebbp*^*Fl/Fl*^*::lsl-MYCN* mice was significantly reduced (approximately 80% within 8 months) compared to the control group (Fig. 1B, SFig. 1A), as those mice had to be sacrificed due to abnormalities in their fur and (brain) tumor-related symptoms (gait abnormality, unwillingness to move, hunched posture, weight loss).

Histological analysis revealed that the forebrain tumors of *hGFAP-cre::Crebbp*^*Fl/Fl*^*::lsl-MYCN* mice consisted of small, round, densely packed cells, which contain pleomorphic nuclei appearing blue in H&E staining (Fig. 1C, D). Tumor cells proliferated strongly, shown by expression of Ki67 (Fig. 1E). Thus, the tumors resemble the histological phenotype used by the WHO to classify tumors of embryonic origin [17]. The tumor cells did not express CREBBP, but strongly expressed MYCN as expected based on their genotype (Fig. 1F, G).

**Fig. 2:**
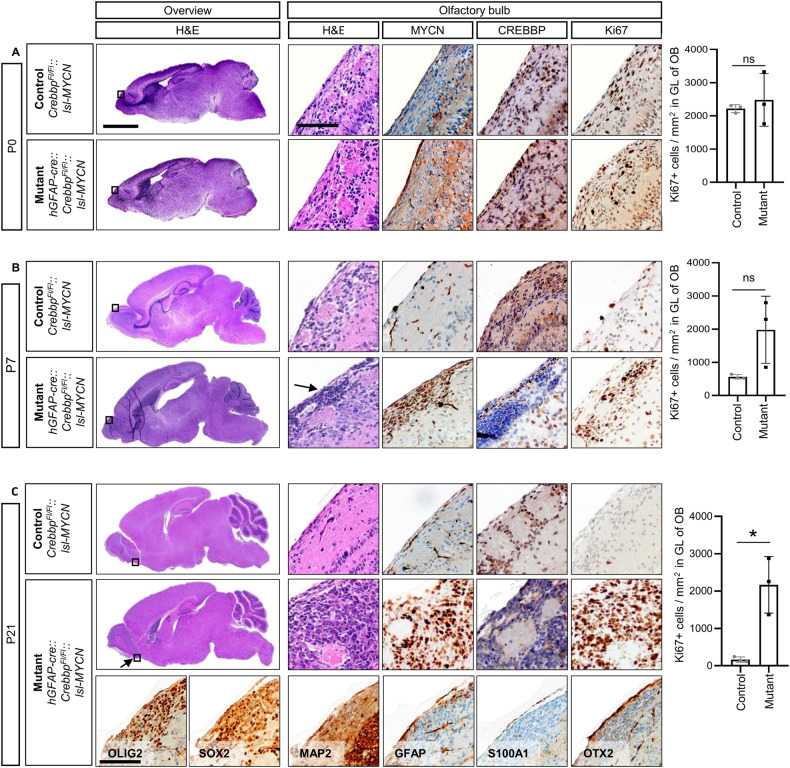
Olfactory bulb tumors are detectable as early as postnatal day 7 in *hGFAP-cre::Crebbp*^*Fl/Fl*^*::lsl-MYCN* mice. **A** Histology of brains of P0 mice (*Crebbp*^*Fl/Fl*^*::lsl-MYCN*, *n* = 3; *hGFAP-cre::Crebbp*^*Fl/Fl*^*::lsl-MYCN*, *n* = 3) including overview images, high power images and IHC of olfactory bulb of P0 mice. No difference in the number of proliferating cells in the periglomerular layer of the OB was observed (*p* = 0.6002). **B** Histology of brains of P7 mice (*Crebbp*^*Fl/Fl*^*::lsl-MYCN*, *n* = 3; *hGFAP-cre::Crebbp*^*Fl/Fl*^*::lsl-MYCN*, *n* = 5) including overview images, high power images and IHC of olfactory bulb of P7 mice. The number of proliferating cells in the periglomerular layer of the OB is higher in the mutant (black, *p* = 0.0724). **C** Histology of brains of P21 mice (*Crebbp*^*Fl/Fl*^*::lsl-MYCN*, *n* = 3; *hGFAP-cre::Crebbp*^*Fl/Fl*^*::lsl-MYCN*, *n* = 4) including overview images, high power images and IHC of olfactory bulb of P21 mice. Extended IHC of olfactory bulb tumors of 3-week-old mice: OLIG2, SOX2, MAP2, GFAP, S100A1, OTX2. The number of proliferating cells in the periglomerular layer of the OB is clearly increased in the mutant (black, *p* = 0.0103). Boxes mark area of high power images. Arrows mark the tumor. Scale bar corresponds to 2 mm (overview) and 100 μm (high power images). *p < 0.05; ns, *p* > 0.05; unpaired *t* tests; vertical bars: SD.

We then studied the mice for early signs of tumor growth throughout postnatal development, aiming to gain knowledge about the time course and exact origin of tumor formation. In histological analyses of brains of newborn mice (postnatal day 0: P0) we found no differences, i.e. no tumors and no abnormal proliferation (Ki67) or accumulation of undifferentiated cells between *hGFAP-cre::Crebbp*^*Fl/Fl*^*::lsl-MYCN* brains and brains of the control littermates (*Crebbp*^*Fl/Fl*^*::lsl-MYCN*) (Fig. 2A, SFig. 1B, C). On P7, transgenic animals developed small tumors in the glomerular layer, the outermost layer of the OB (Fig. 2B). These early lesions proliferated strongly, and MYCN staining confirmed overexpression in the tumor. Depletion of CREBBP was verified by corresponding staining (Fig. 2B). Brains of 21-day-old mice showed that the OB tumors grow fast and proliferate strongly (Fig. 2C). CREBBP and MYCN stainings of OBs of P21 mice confirmed strong accumulation of MYCN and loss of CREBBP in tumor tissue compared to the control. Further immunohistochemical staining did not yield a clear classification of the tumors regarding either neural or astroglial origin: The early tumor cells (P21 mice) were positive for oligodendrocyte lineage marker OLIG2 and SOX2, a marker for CNS progenitor cells, e.g. NSCs. Moreover, they showed low expression of the dendritic marker MAP2 and stained negative for astrocyte markers GFAP and S100A1 as well as for OTX2, a marker for dopaminergic neurons.

In addition to the OB tumors, several diffusely distributed proliferating cells were noted in *hGFAP-cre::Crebbp*^*Fl/Fl*^*::lsl-MYCN* mice. These cells were detected in the mid- and hindbrain and were associated with blood vessels or the brain surface (SFig. 1D–F). In contrast to the OB tumors, the diffuse cell clusters did not grow as large, solid tumors in adult mice. Marker expression of CREBBP, MYCN, GFAP, MAP2, SOX2, and S100A1 resembled the expression in the OB tumors (SFig 1E–G/data not shown). However, cells in the mid- and hindbrain regions expressed OTX2, but not OLIG2, suggesting that these are independent lesions developing in parallel to the forebrain tumors (SFig. 1G/data not shown).

Of note, previously generated transgenic mice, which carry a loss of *Crebbp* (*hGFAP-cre::Crebbp*^*Fl/Fl*^) or an overexpression of *MYCN* (*hGFAP-cre::lsl-MYCN*) do not develop brain tumors [8, 19]. In *hGFAP-cre::Crebbp*^*Fl/Fl*^ mice, impaired neural precursor cell differentiation and migration lead to structural abnormalities of the brains, e.g. in the OB. Mice carrying only the MYCN transgene (*hGFAP-cre::lsl-MYCN)* show pancreas and pituitary gland tumors derived from neuroendocrine *hGFAP*-expressing cells in the two organs. As expected, we also observed such tumors in our new mouse model with a comparable frequency and latency. Taken together, we demonstrated tumor development in the murine OB with early postnatal onset induced by the cooperative effects of *Crebbp* inactivation and overexpression of exogenous human *MYCN*.

### Characterization of *hGFAP-cre::Crebbp*^*Fl/Fl*^*::lsl-MYCN* tumor cells

**Fig. 3:**
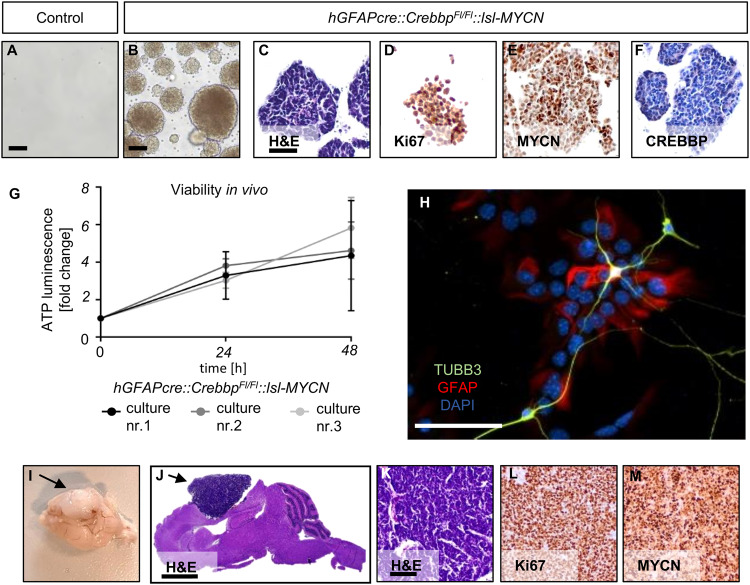
*hGFAP-cre::Crebbp*^*Fl/Fl*^*::lsl-MYCN* tumor cells display stem cell features in culture and form tumors in recipient mice. **A**, **B** Cultivation of *hGFAP-cre::Crebbp*^*Fl/Fl*^*::lsl-MYCN* tumor cells (**B**) and control tissue (**A**) in NSC Medium. *N* = 5. Scale bar corresponds to 50 μm. **C**–**F** High power images and IHC of tumorspheres. Scale bar corresponds to 50 μm. **G** Viability assay showing in vitro growth of three newly established *hGFAP-cre::Crebbp*^*Fl/Fl*^*::lsl-MYCN* tumor cell lines (nr.1-3) (*n* = 3, experiments performed in triplicates). Vertical bars: SD. **H** IHC of in vitro differentiated *hGFAP-cre::Crebbp*^*Fl/Fl*^*::lsl-MYCN* tumor cells. Staining for neural (TUBB3) and glial (GFAP) lineage markers. Scale bar corresponds to 50 μm **I** Macroscopic image of tumors in recipient mouse brain after tumor cell transplantation. Two of 9 mice developed tumors. **J** Histological overview image of recipient mouse brain. Scale bar corresponds to 2 mm. **K**–**M** High power images and IHC of recipient mouse tumor. Scale bar corresponds to 100 μm. Arrows mark tumors.

With the aim to further investigate characteristics of the tumor cells, we isolated and dissociated OB cells of 3- to 4-week-old *hGFAP-cre::Crebbp*^*Fl/Fl*^*::lsl-MYCN* mice and their *Crebbp*^*Fl/Fl*^*::lsl-MYCN* littermates. The cells were cultured in serum-free medium with growth factors (NSC medium) for two weeks. While there was no cell growth in the control (Fig. 3A), cells from *hGFAP-cre::Crebbp*^*Fl/Fl*^*::lsl-MYCN* mice grew as tightly packed spheres that stained positive for the proliferation marker Ki67 and the oncoprotein MYCN, but negative for CREBBP (Fig. 3B–F). Therefore, the spheres are most likely composed of tumor cells.

In cultures from *hGFAP-cre::Crebbp*^*Fl/Fl*^*::lsl-MYCN* tumor cells (*n* = 3) the number of viable cells increased over time, as demonstrated by a corresponding CellTiter-Glo^®^ Luminescent Cell Viability in vitro assay (Fig. 3G). Upon removal of growth factors and the addition of serum, the spheres differentiated into adherent cells expressing the glial lineage marker GFAP and the neuronal lineage marker TUBB3 [34] (Fig. 3H), illustrating that the sphere-forming cells maintain differentiation potential towards various lineages.

Next, sphere-forming tumor cells were transplanted into the forebrain of CD1 nu/nu mice in order to examine the neoplastic potential of these cells. Two of 9 mice showed brain tumour-related symptoms at approximately 13 weeks post-transplantation. These mice developed tumors in the injection area (Fig. 3I, J). Tumors were analyzed histologically and resembled the phenotype of the primary *hGFAP-cre::Crebbp*^*Fl/Fl*^*::lsl-MYCN* tumors (Fig. 3K–M).

Hence, *hGFAP-cre::Crebbp*^*Fl/Fl*^*::lsl-MYCN* tumor cells can be characterized as proliferative, multipotent, and tumorigenic in the forebrain (non-olfactory bulb) microenvironment.

### OB tumors from *hGFAP-cre::Crebbp*^*Fl/Fl*^*::lsl-MYCN* mice show high intratumoral cell diversity and most likely originate in the V-SVZ stem cell niche

**Fig. 4:**
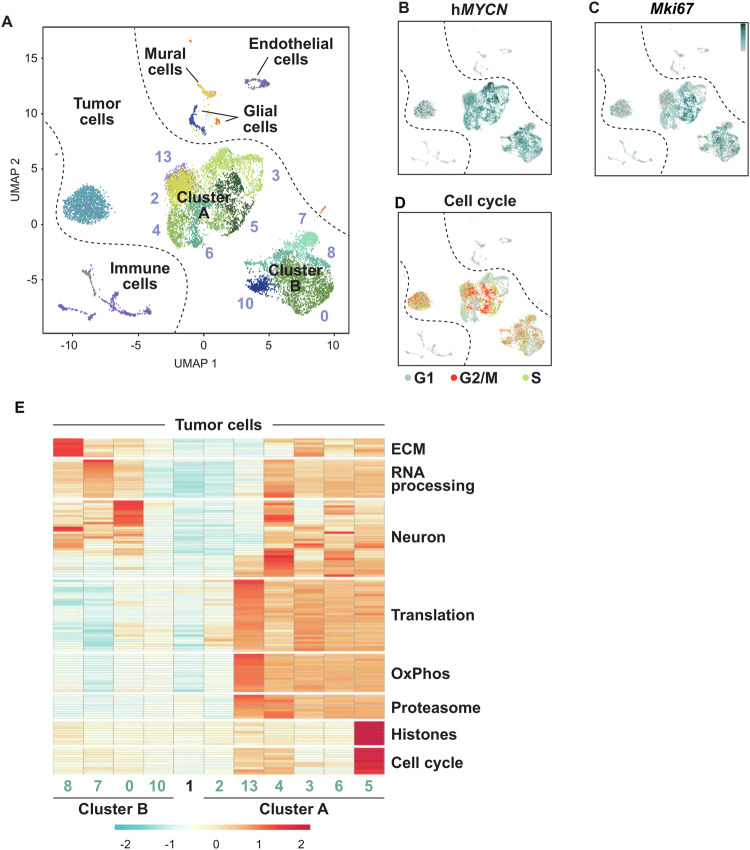
Intratumoral heterogeneity of *hGFAP-cre::Crebbp*^*Fl/Fl*^*::lsl-MYCN* tumors as revealed by scRNA-seq. **A** UMAP plot showing clustering and cell class annotation for single-cell transcriptomes from *hGFAP-cre::Crebbp*^*Fl/Fl*^*::lsl-MYCN* cells. **B**, **C** Relative expression values (log2) of human MYCN (hMYCN (**B**)) and the proliferation marker *Mki67* (**C**), cells without marker detection are in gray. **D** Expression of cell cycle phase (G1, G2/M, S) signature genes in individual cells of *hGFAP-cre::Crebbp*^*Fl/Fl*^*::lsl-MYCN* tumors. **E** Heatmap showing the expression of distinct functional gene classes in all tumor cell clusters. ECM, extracellular matrix; OxPhos, oxidative phosphorylation.

To gain a deeper insight into the cellular composition and origin of these malignant OB tumors, we used droplet-based single-cell RNA sequencing (scRNA-seq) of two tumors from *hGFAP-cre::Crebbp*^*Fl/Fl*^*::lsl-MYCN* mice. After quality control and elimination of batch effects, 12,234 single-cell transcriptomes were included in the final dataset. A total of 17 clusters were resolved (Fig. 4A). The cells of both individual tumors were distributed between all clusters, although not evenly (SFig. 2A). By analyzing the differentially expressed genes (DEG) in the individual clusters and by including cell type-specific marker genes for defined cell populations from a comprehensive reference single-cell atlas of the developing mouse brain [35], it was possible to distinguish tumor cells from non-malignant cells. The latter form the tumor microenvironment (TME) and accounted for 11% of the total cells. The TME cells could be divided into various clusters including defined immune cell populations and stromal cells (SFig. 2B–E).

However, tumor cells made up the majority of the cells, namely 89%. Notably, apart from the "isolated" Cluster 1, all other tumor cell subpopulations were combined into two larger, superordinate clusters: Cluster A with 6 and Cluster B with 4 individual clusters. In the following, we focused on further characterizing the similarities and differences of these tumor cell subpopulations. A high intratumoral cell heterogeneity was visible on several levels. All tumor cell populations showed high expression levels of the human MYCN (h*MYCN*) reporter gene (Fig. 4B). However, for example, the h*MYCN* expression pattern was not very strongly correlated with that of the proliferation marker Ki67 (*Mki67*) (Fig. 4C). The latter was almost identical to that of cells in the G2/M cell cycle phase; but here, too, clear differences were recognizable within individual subpopulations of the tumor cells (Fig. 4D). DEG and gene ontology (GO) analyses revealed further differences in the gene expression programs of the two Meta-Clusters, as can be seen in the associated heatmap (Fig. 4E; SFig. 3). For example, they were clearly separated by gene programs coupled to cellular metabolism, such as protein biosynthesis/translation, oxidative phosphorylation (OxPhos), and proteasomal activity, which were highly expressed in Cluster A cells. Interestingly, there was also a clear dichotomy between Cluster A and B cells in terms of neuro-specific expression patterns. Many cells of Cluster B, particularly those in Cluster 0, showed high expression of several markers for mature neurons; examples are the glutamate receptor gene *Grin1* and *Gpm6a*, which encodes a brain-specific glycoprotein placed at the surface of neurons [36]. In contrast, Cluster A cells contained several highly expressed genes with neurodevelopmental functions that are important for the differentiation of progenitor cells into more mature neuronal stages. Examples are the microtubule-associated gene *Map1b*, which is expressed in the developing neural tube [37], and the homeobox gene *Dlx5*, which is essential for neurogenesis of olfactory neurons [38].

**Fig. 5:**
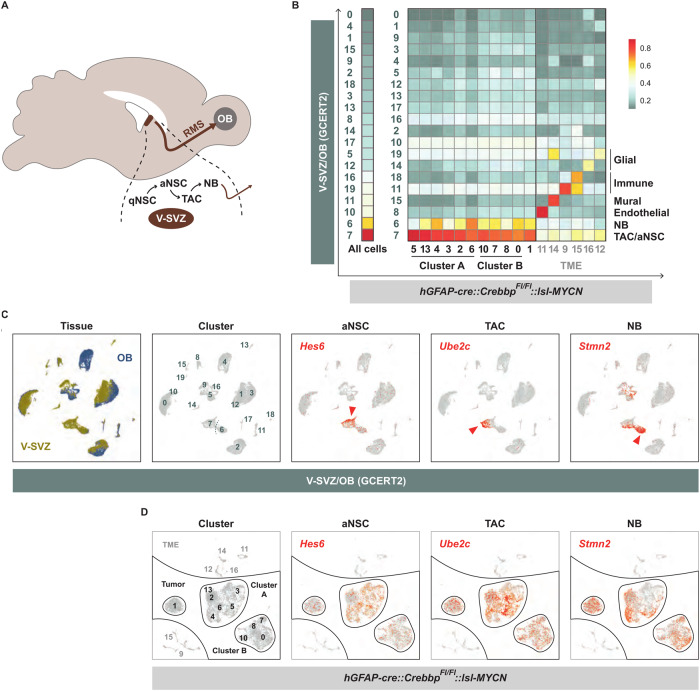
OB tumors of *hGFAP-cre::Crebbp*^*Fl/Fl*^*::lsl-MYCN* mice originate from the V-SVZ stem cell niche. **A** Schematic illustration showing the location of the subventricular zone of the lateral ventricle wall (V-SVZ) and the olfactory bulb (OB) in the adult mouse brain. Quiescent neural stem cells (qNSC), actively dividing NSCs (aNSC) and transition-amplifying cells (TAC) are located in the V-SVZ and give rise to neuroblasts (NB) that migrate through the rostral migratory stream (RMS) into the olfactory bulb. **B** Similarity scores of murine transgene tumors and cell types of the adult ventricular-subventricular zone and olfactory bulb region (V-SVZ/OB) in the GCERT2 fate mapping model (ref. [23]). Similarity was calculated by logistic regression, either for all tumor cells together (left), or separately for individual clusters (right). Colors represent the probability of high (red) to low (gray) similarity. **C**, **D** UMAP plots showing tissue and cluster distribution of GCERT V-SVZ/OB cells (**C**), of *hGFAP-cre::Crebbp*^*Fl/Fl*^*::lsl-MYCN* cells (**D**), and expression of marker genes for aNSC (*Hes6*), TAC (*Ube2c*), and NB (*Stmn2*) in both datasets. Arrowheads mark cell populations with the highest log2 expression values.

To elucidate the origin of the tumor cells, a similarity analysis was conducted with a reference dataset containing single-cell transcriptomes of cells from the V-SVZ and OB region of adult mice [24]. It comprises data from two independent murine fate-mapping models, *hGFAP::CreER*^*T2*^*; Rosa26*^*LSL-TdTomato*^ (GCERT2) and *ratNes::FLPOER; Rosa26*^*FSF-TdTomato*^ (NESFLPO), which both track adult neuronal lineage progression from quiescent NSCs in the V-SVZ to diverse OB neurons via actively dividing NSCs (aNSCs), transit-amplifying cells (TACs), and neuroblasts (NBs) as intermediates (Fig. 5A). Similarities between tumor and reference cells were calculated using a logistic regression approach. TACs/aNSCs emerged as the cell type with the highest similarity score to the tumor cell population as a whole as well as to each individual subpopulation; this was true for both the GCERT2 model (Fig. 5B) and the NESFLPO model (SFig. 4). A (albeit much weaker) resemblance of the tumor cells to neuroblasts was also observed. TME cells successfully matched corresponding cell types from the reference data set, but showed no similarity to any of the cell types from the V-SVZ/OB stem cell niche (Fig. 5B; SFig. 4A). Intratumoral heterogeneity was also evident in this analysis as for example characteristic marker genes for aNSCs (*Hes6*), TACs (*Ube2c*) and NBs (*Stmn2*) (Fig. 5C) are expressed to different extents in the different tumor cell populations (Fig. 5D).

In summary, the tumors from our mouse model show a broad cellular diversity. Based on our transcriptomics study, clear differences between tumor subpopulations can be identified with regard to their neurodevelopmental status and their metabolic and proliferative activity. Due to the overall high similarity of these cells with stem/progenitor cells from the V-SVZ stem cell niche, the latter most likely represent the common cell of origin of *hGFAP-cre::Crebbp*^*Fl/Fl*^*::lsl-MYCN* tumors.

### V-SVZ derived NSCs from *hGFAP-cre::Crebbp*^*Fl/Fl*^*::lsl-MYCN* mice display altered viability, proliferation, and differentiation potential in vitro

We next investigated the V-SVZ of *hGFAP-cre::Crebbp*^*Fl/Fl*^*::lsl-MYCN* mice as cells within this region were suggested as the origin for the OB tumors. There were no major histological differences detected between the V-SVZ regions of control and mutant mice in sagittal sections of P0, P7, P21 or in frontal sections of P7 mice (SFig. 5/data not shown).

**Fig. 6:**
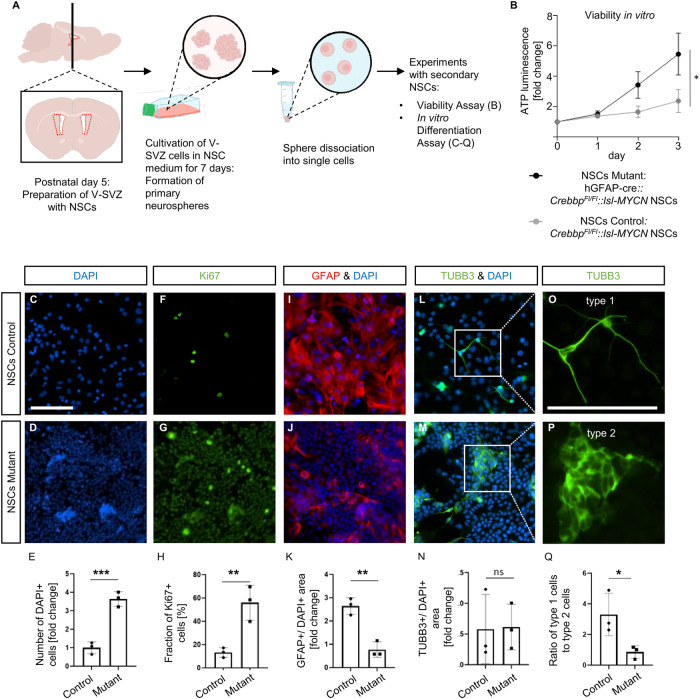
Subventricular NSCs display abnormal proliferation and differentiation potential. **A** Schematic image of NSC extraction. **B** Viability assay showing in vitro growth of *hGFAP-cre::Crebbp*^*Fl/Fl*^*::lsl-MYCN* NSCs versus *hGFAP-cre NSCs* (Control), *n* = 3, experiments performed in triplicates. *, p < 0.05 (p = 0.0274); Unpaired *t* test of day 3. Vertical bars: SD. **C**–**Q** IHC of in vitro differentiated *hGFAP-cre* NSCs and *hGFAP-cre::Crebbp*^*Fl/Fl*^*::lsl-MYCN* NSCs. Both scale bars correspond to 100 μm. **C**–**E** Representative images (**C**, **D**) and fold change of DAPI positive cells (**E**, *p* = 0.0009). **F**–**H** Representative images (**F**, **G**) and fold change of Ki67 positive cells (**H**, *p* = 0.009). **I**–**K** Representative images of GFAP and DAPI positive cells (**I**, **J**) and fold change of GFAP positive area (**K**, *p* = 0.0027). **L**–**N** Representative images of TUBB3 and DAPI positive cells (**L**, **M**) and fold change of TUBB3 positive area (**N**, p = 0.93). **O**–**Q** Magnification of (L) and (M) (**O**, **P**) and ratio of number of phenotypically type 1 to phenotypically type 2 TUBB3+ cells (**Q**, *p* = 0.04). *N* = 3. Ten pictures were taken from each experiment. *p < 0.05; **p < 0.01; ***p < 0.001; ns, *p* > 0.05; unpaired *t* tests; vertical bars: SD.

Moreover, in vitro characteristics of secondary NSCs were analyzed to further examine relationships between tumor cells and cells from the stem cell niche in our mouse model. NSCs were extracted from the V-SVZ of five-day-old mice. They were cultured for 7 days in NSC medium, and then split into single cells (Fig. 6A). NSCs from transgenic mice displayed a significantly higher viability compared to control NSCs (Fig. 6B). Furthermore, in vitro differentiation assays indicated significant differences between NSCs from transgenic mice and control animals. The number of differentiated NSCs after 7 days in culture with serum, but without growth factors, was assessed by counting DAPI stained nuclei and was significantly higher than in controls (Fig. 6C–E). Furthermore, the fraction of proliferating cells was higher, determined after DAPI and Ki67 double staining (Fig. 6F–H). Differences in cellular differentiation were evident in the expression of cell-lineage markers. NSCs from transgenic mice exhibited reduced glial differentiation compared to control NSCs, measured as proportion of the area of cells positive for the glial marker GFAP to DAPI stained area (Fig. 6I–K). Even though the proportion of area staining positive for the neural lineage marker TUBB3 to DAPI positive area did not significantly differ between control and mutant NSCs (Fig. 6L–N), phenotypic differences were noticed (Fig. 6O, P). Type 1 cells resembled mature neurons and generated axons, while type 2 cells—abundant in transgenic mice—grew in aggregates and appeared less differentiated without neuronal processes. In the control group, a larger fraction of TUBB3 positive cells showed type 1 features, whereas in the mutant group, the ratio of type 1 cells to less differentiated type 2 TUBB3 positive cells was significantly shifted towards the latter (Fig. 6Q). Together, their high proliferation rate and impaired differentiation potential in addition to the previously described findings from scRNA sequencing suggest that NSCs from the V-SVZ of *hGFAP-cre::Crebbp*^*Fl/Fl*^*::lsl-MYCN* mice represent a pre-tumorigenic cell type closely related to the OB tumor cells.

## Discussion

Genetically engineered mice combining depletion of CREBBP and overexpression of *MYCN* in NSCs (*hGFAP-cre::Crebbp*^*Fl/Fl*^*::lsl-MYCN*) presented with forebrain tumors arising in the OBs with 75% penetrance. Tumor-derived cell lines proliferated strongly both in vitro and as allografts in immunocompromised mice, with 2 of 9 recipient mice developing tumors, confirming neoplastic transformation/acquisition of neoplastic properties. In contrast, CREBBP loss or *MYCN* overexpression alone in *hGFAP*-positive cells does not induce malignant brain tumors, as previously reported [8, 19], indicating that tumorigenesis in *hGFAP-cre::Crebbp*^*Fl/Fl*^*::lsl-MYCN* mice depends on concurrent alterations in both genes.

When solitary *MYCN* expression is driven under the control of the *hGFAP* promoter [19], transgenic mice (*hGFAP-cre::lsl-MYCN*) develop neuroendocrine tumors of the pancreas and pituitary adenomas. These tumors were recapitulated in *hGFAP-cre::Crebbp*^*Fl/Fl*^*::lsl-MYCN* mice with a comparable frequency and latency if mice were not sacrificed due to brain tumor symptoms before non-CNS tumor development. Neuroendocrine tumors arose in around 60% of *hGFAP-cre::lsl-MYCN* mice with two extra copies of *MYCN*, mostly between 6 and 11 months of age, while mice with only one additional copy of exogenous *MYCN* stayed tumor-free (SFig. 1A, ref. [28]). In contrast, *hGFAP-cre::Crebbp*^*Fl/Fl*^*::lsl-MYCN*^*Fl/Wt*^ *mice* with one additional copy of exogeneous *MYCN*, also developed brain tumor although the penetrance for brain tumors was slightly lower (approx. 25% in 6 months) (SFig. 1A). The respective penetrance of brain tumors and neuroendocrine tumors in both *MYCN*-based models (*hGFAP-cre::Crebbp*^*Fl/Fl*^*::lsl-MYCN* and *hGFAP-cre::lsl-MYCN*) was most likely dependent on *MYCN* dosage. Together with the observation that MYCN levels were undetectable or low in target cells before first occurrence of OB tumors and increased with tumor growth, the incomplete penetrance might be explained by variability of MYCN accumulation, which does not always reach the threshold required for tumor development.

Mice with a knockout of *Crebbp* in hGFAP-positive cells (*hGFAP-cre::Crebbp*^*Fl/Fl*^) do not develop any tumors [8], however they show non-neoplastic cell aggregations close to the V-SVZ, which are preserved in the *hGFAP-cre::Crebbp*^*Fl/Fl*^*::lsl-MYCN* mouse model.

These aggregations are due to a migration deficit of neuroblasts, potentially mediated by IGF1 signaling [8]. Recently, Chen et al. reported that olfactory stimuli can support brain tumor formation in the rodent OB and implied a central role of IGF1 expressed by OB projection neurons [39]. We suppose that early postnatal tumor formation in *hGFAP-cre::Crebbp*^*Fl/Fl*^*::lsl-MYCN* mice might be influenced by onset of olfactory experience soon before, and/or by structural and functional disturbances of OB neurons related to CREBBP loss, given that growing evidence indicates neuronal activity to influence tumorigenesis in brain and other tissues [10]. Our model system could be valuable to further explore links between olfactory input, altered neuronal function and tumorigenesis.

Given the location of the tumors in the OB and their early onset, a connection of tumorigenesis to physiological OB development, with V-SVZ NSCs as cells of origin for the tumors, appears likely.

Pre- and postnatal development of the OBs in mice is complex and not yet completely understood [22]. Virtually all OB projection neurons are generated prenatally from local progenitors in the OB germinal zone. Interneurons greatly outnumber projection neurons in the mature OB. While some interneurons are generated during embryonic development, most arise during the first few postnatal weeks and later continue to be produced via adult neurogenesis in the V-SVZ from where they migrate into the OB via the RMS [22]. This V-SVZ-RMS-OB-axis involved in lifelong adult neurogenesis in mice makes it plausible that tumor formation has been observed in the OBs in many brain cancer mouse models [34, 39].

During embryonic development, OB interneurons originate from diverse germinal regions including the ganglionic eminences, pallium and septum, which later form the V-SVZ. A minor fraction of interneurons emerges from endogenous embryonic OB precursor cells, at least until early postnatal life. Moreover, immature precursor cell populations were identified in OB scRNA sequencing studies [40]. With this knowledge, it cannot be ruled out that the OB tumors in *hGFAP-cre::Crebbp*^*Fl/Fl*^*::lsl-MYCN* mice arose from local progenitors. However, the peak of interneuron generation in the OB is within the first weeks of life [41], which coincides with tumor development in our mouse model. These neurons are generated in the SVZ, migrate through the RMS into the OB, and make up most of the cells of the OB. The timing and location of the tumor development at P7 between the periglomerular interneurons of the OB therefore raised the question whether they might originate from the V-SVZ stem/progenitor cell compartment.

As an independent approach to investigate cellular characteristics of the OB tumors and their presumptive roots in the V-SVZ stem cell niche, we used single-cell RNA sequencing and performed a comparison with reference cell types of the adult V-SVZ and OB region [24]. Despite uniform appearance on the histological level, this analysis revealed considerable intratumoral heterogeneity on transcriptional level with three main clusters of tumor cells. The two major clusters differ markedly in their gene expression related to metabolic and proliferative activity and to neuronal properties. Although the different subpopulations most closely resemble different progenitor cell types, all cells overall show high similarity to stem/progenitor cells from the V-SVZ niche.

In comparison to adult V-SVZ / OB reference cells, we obtained TACs / aNSCs as the cell type with the highest similarity score to all tumor cells. These activated cell types arise from qNSCs in dormant cell state in the V-SVZ of adult mice [21]. The majority of later qNSCs was shown to originate from specific radial glia populations as progenitors, transit to a quiescent state from mid-embryonic development and remain largely quiescent until at least young adulthood [23]. Recent data suggests that the embryonal progenitors of qNSCs show transcriptional similarity with adult aNSCs [26], implying potential conversions between active and dormant cell states instead of fundamental changes in cell identity. Thus, this continuum of cells in the V-SVZ stem cell niche likely contains the cell of origin of OB tumors in *hGFAP-cre::Crebbp*^*Fl/Fl*^*::lsl-MYCN* mice.

Therefore, we examined the properties of V-SVZ NSCs derived from transgenic mice at young age. Whereas there was no histologic evidence of hyperplasia or increased proliferation in the V-SVZ in vivo, mutant V-SVZ NSCs showed aberrant growth with increased cell viability and proliferation as well as altered differentiation potential with decreased glial and neural differentiation compared to control cells in vitro. The ability of MYCN to transform NSCs has been described before [29]. However, in their study only mutationally stabilized MYCN but not wild type MYCN was able to increase proliferation and change the differentiation behavior of NSCs. With our model, we are presenting a second tumorigenic hit, which allows tumor formation with a wild-type MYCN, possibly by transcriptional or epigenetic changes allowing the accumulation of MYCN in the cells.

Mounting evidence implicates V-SVZ NSCs as potential cells of origin in the pathogenesis of human glioblastoma [42–44]. Animal models have corroborated such connections, demonstrating migratory patterns of cancer-forming NSC-derived cells from the V-SVZ to tumors [42–45]. Of note, in a pan-genomic screen, *CREBBP* was identified to be among just five genes (comprising also *TP53*), in which mutations reproducibly caused a significant proliferative advantage in isolated human NSCs [46]. Though we did not formally proof a direct clonal relationship to OB tumors, mutant V-SVZ NSCs might represent a pre-tumorigenic cell population as indicated by their abnormal growth and differentiation patterns.

On the other hand, we performed a comparison of histological markers and copy number variations of human ONBs and our mouse tumors, as olfactory neuroblastoma (ONB) are human brain tumors, which typically arise in the human olfactory bulb. However, mouse tumors did not resemble key aspects of human ONBs as expected (Fig. S6).

Attempts to match the OB tumors to human tumor entities by bulk and single-cell transcriptome analyses remained inconclusive (data not shown), but similarities to specific human tumors might emerge when more data for specific subgroups of human tumors or tumor cell subpopulations become available. In order to better understand the cooperation between CREBBP and MYCN, especially in the human system, further experiments will be needed that determine whether similar effects and gene expression patterns are conserved in human stem cells.

Taken together, our study adds to the multi-faceted views of brain cancers as developmental diseases, recognizing tumor cells as locked in dysregulated cellular states of developmental cell populations and identifying impaired differentiation of specific neural progenitors as common mechanism in pediatric neurogenic cancers [10, 47–50]. We conclude that combined loss of CREBBP and overexpression of *MYCN* in NSCs induces brain tumor formation with early postnatal manifestation and specific location in the OB. These combined alterations set a framework to a wide range of neural stem/progenitor cells in the developing mouse brain, which in vulnerable cells, presumably within the V-SVZ stem cell compartment, favors distortion of subsequent development resulting in tumorigenesis. *hGFAP-cre::Crebbp*^*Fl/Fl*^*::lsl-MYCN* mice can serve as a model to unravel pathogenetic mechanisms in brain tumors with simultaneous aberrations of *CREBBP* and *MYCN*, such as exemplifying broad dysregulation of gene expression by concurrent deviations of a chromatin modifier and global transcriptional regulator and a member of the MYC family.

## Supplementary information

Suppl. Figures

## Data Availability

The sequencing data and count matrices from the *hGFAP-cre::Crebbp*^*Fl/Fl*^*::lsl-MYCN* mouse tumors reported in this paper are available at Gene Expression Omnibus (GEO) under accession code GSE214983. The accession number for the data from Mizrak et al. [24] used for logistic regression analysis is GEO: GSE134918.
