# Supplementary material for: The tumor suppressor CREBBP and the oncogene MYCN cooperate to induce malignant brain tumors in mice
Source: Oncogenesis. 2023 Jul 5;12(1):36. doi: 10.1038/s41389-023-00481-3 (PMC10322855; doi:10.1038/s41389-023-00481-3)
Supplement: Supplementary file 1 — Suppl. Figures [file 41389_2023_481_MOESM1_ESM.pdf]

Supplementary Figure 1

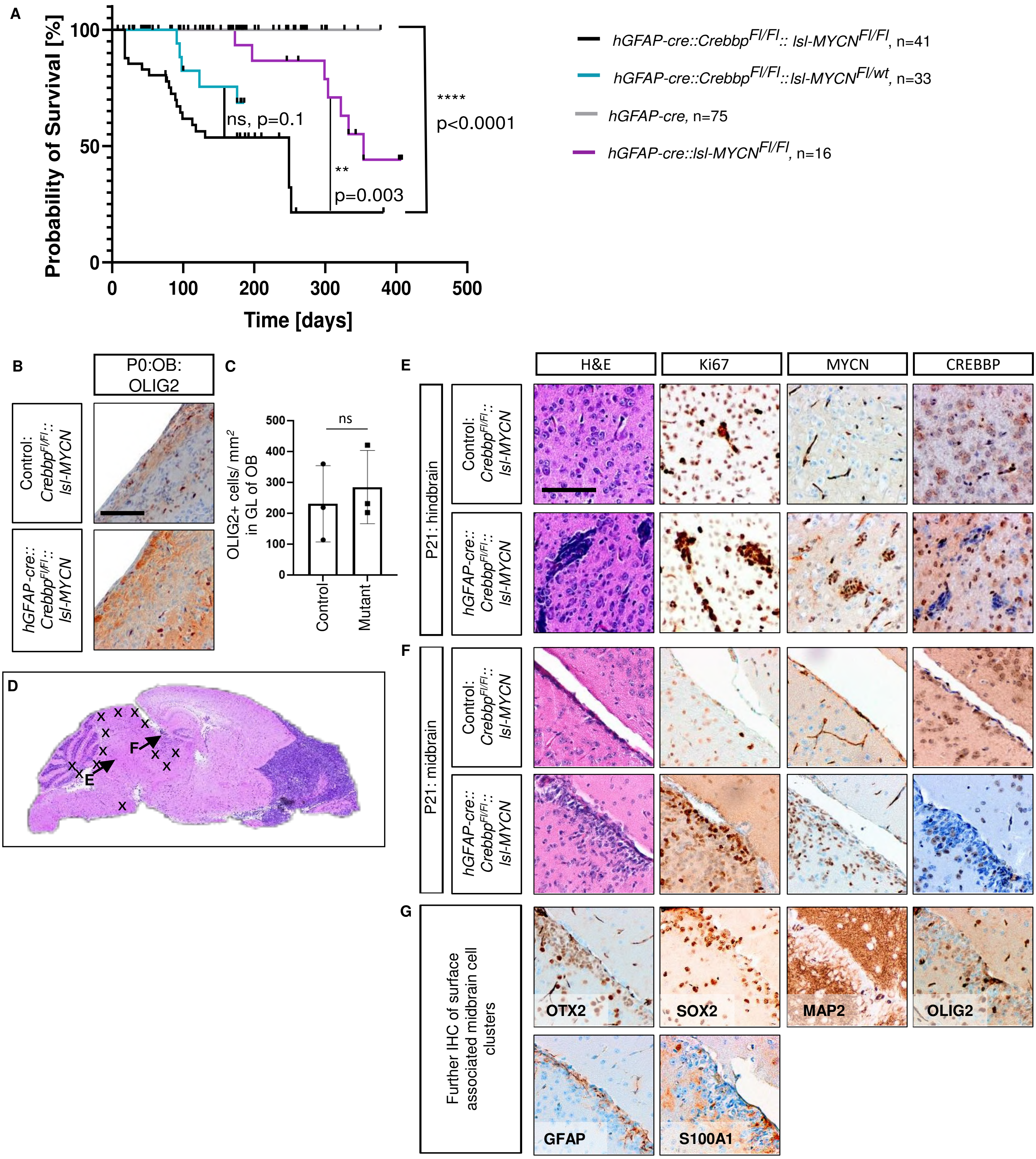

Supplementary Figure 1.

A, Kaplan-Meier survival curves of all possible genotypes. Survival of mice with both transgenes is significantly worse (Log-Rank test) compared to wildtype or animals with only the *MYCN* transgene. The survival difference between mice with one or two alleles of MYCN (*Fl/Fl* vs *Fl/wt*) is not significant. B, OLIG2 staining of OB of P0 mice (*Crebbp*$^{Fl/Fl}$*::lsl-MYCN*, n=3; *hGFAP-cre::Crebbp*$^{Fl/Fl}$*::lsl-MYCN*, n=3). C, Counts of OLIG2 positive cells in the glomerular layer of the OB of P0 mice. ns, p > 0.5. D, Histological overview image of brain of an adult *hGFAP-cre::Crebbp*$^{Fl/Fl}$*::lsl-MYCN* mouse. Arrows mark vessel associated and surface associated cell clusters. Crosses mark mid- and hindbrain areas, where cell clusters were found frequently. E, High power images of vessel associated cell clusters (*hGFAP-cre::Crebbp*$^{Fl/Fl}$*::lsl-MYCN*, n=3) and corresponding region in control hindbrains (*Crebbp*$^{Fl/Fl}$*::lsl-MYCN*, n=3). F, High power images of surface associated cell clusters (*hGFAP-cre::Crebbp*Fl/Fl*::lsl-MYCN*, n=3) and corresponding regions in control midbrains (*Crebbp*$^{Fl/Fl}$*::lsl-MYCN*, n=3). G, Further IHC of surface associated cell clusters. Scale bars correspond to 100 µm.

Supplementary Figure 2

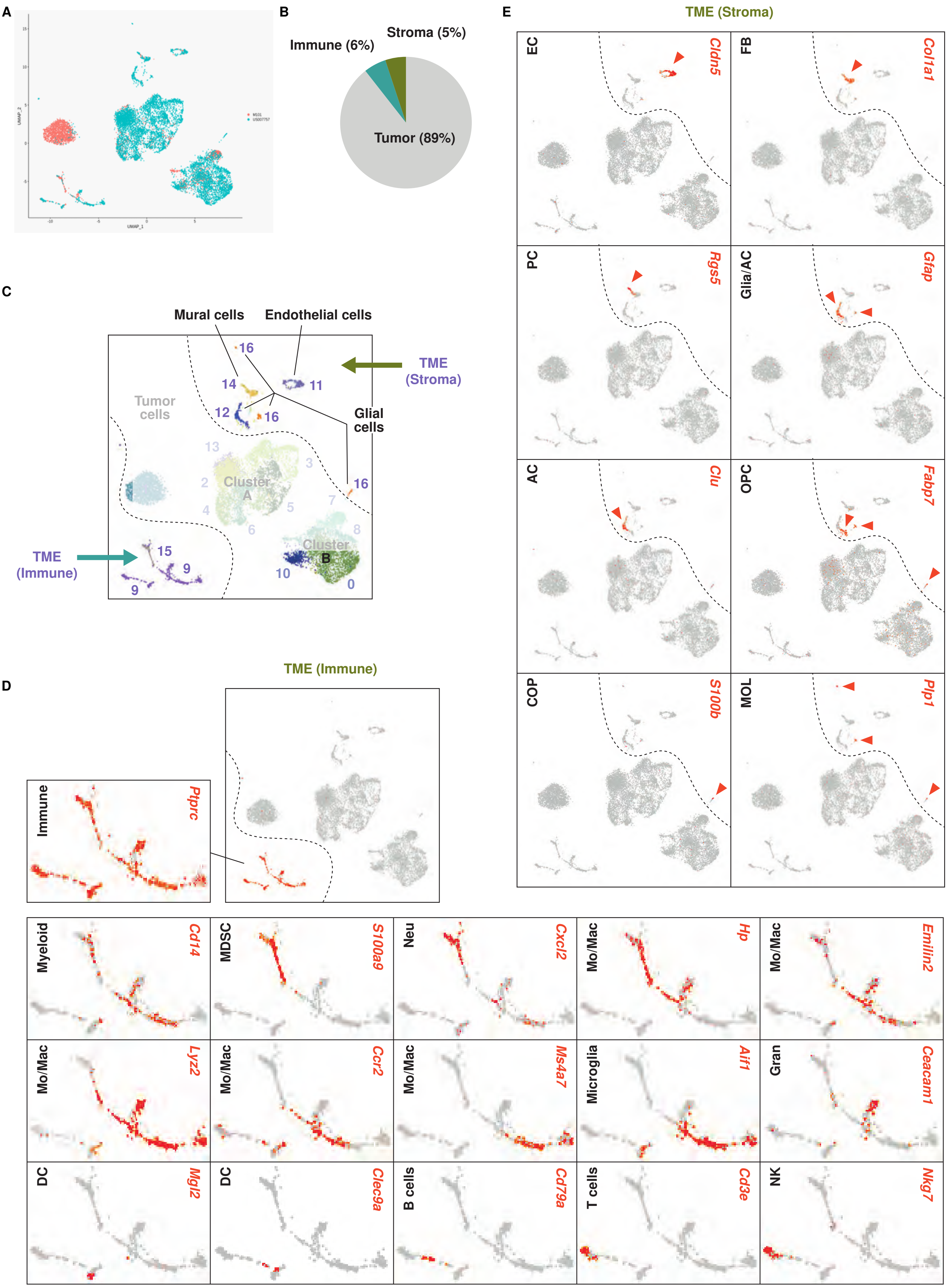

Supplementary Figure 2. A**,** UMAP depicting the origin of tumor cells from tumor 1 (orange) or 2 (turquoise). B**,** Pie chart showing the relative frequencies of tumor cells and of stroma and immune cells in the TME. C**,** UMAP plot with highlighted clusters of TME cells. D**,**E, UMAP plots showing expression levels of different cell type-specific marker genes for tumor-associated stromal cells (D), and for immune cells (E). AC, astrocytes; COP, commited oligodendrocyte precursors; DC, dendritic cells; EC, epithelial cells; FB, fibroblasts; Gran, granulocytes; MDSC, myeloid-derived suppressor cells; MG, microglia; MOL, mature oligodendrocytes; Mo/Mac, Monocytes/Macrophages; Neu, neutrophils; NK, natural killer cells; OPC, oligodendrocyte precursor cells; PC, pericytes.

Supplementary Figure 3

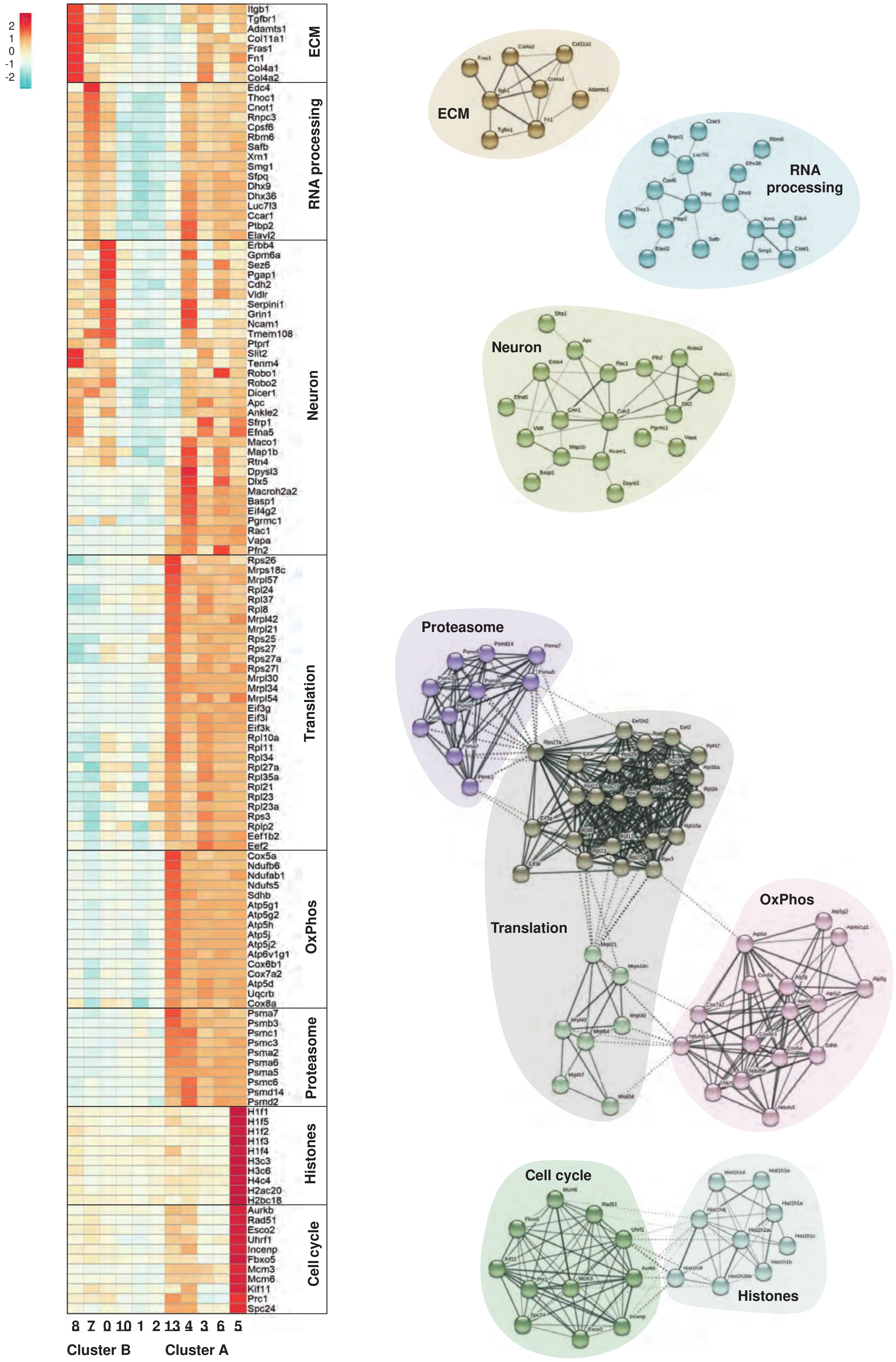

Supplementary Figure 3. Extended view of the heatmap from Figure 4E. Functional networks of the individual gene classes are shown on the right, as revealed by STRING (string-db.org).

Supplementary Figure 4

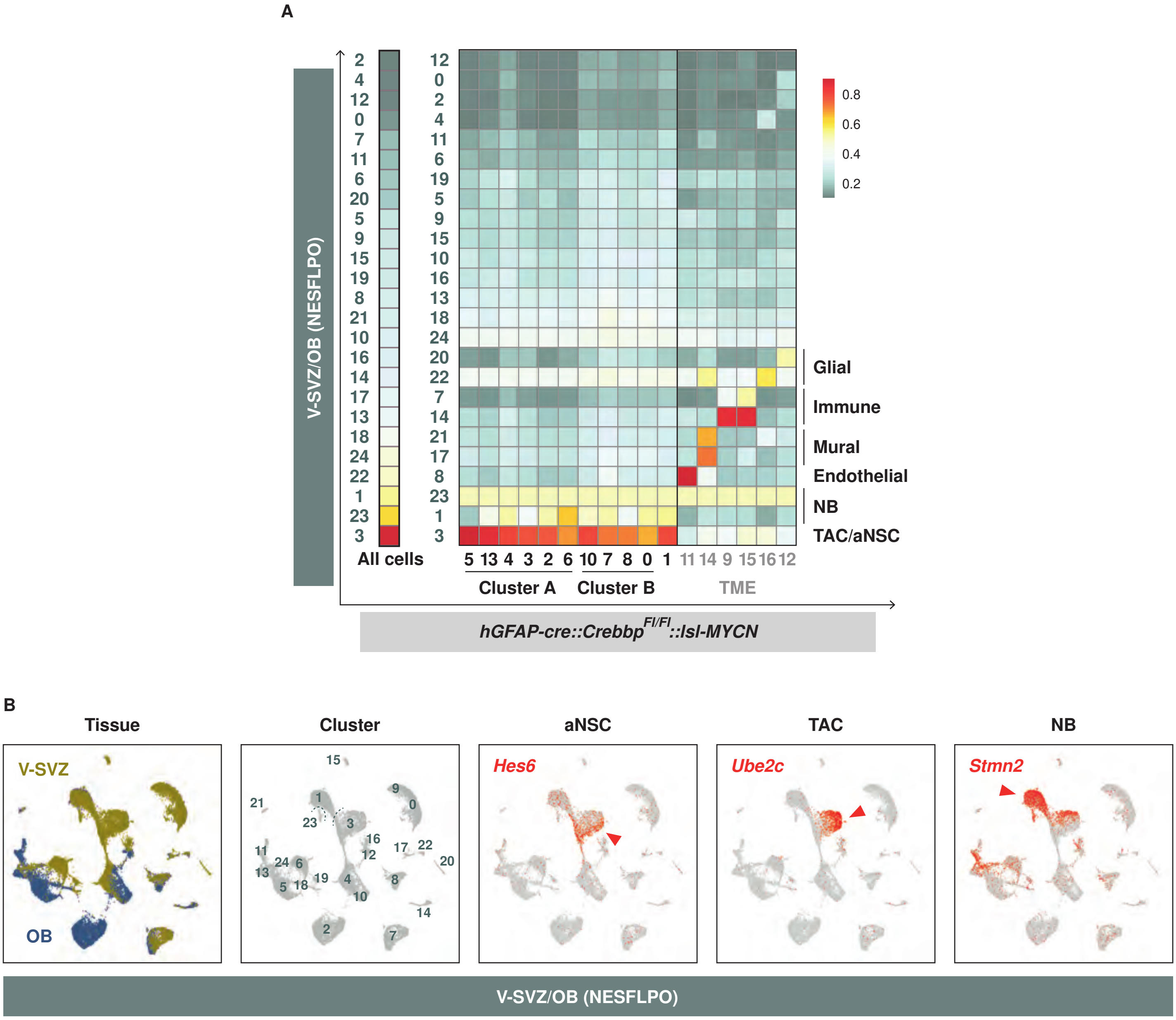

Supplementary Figure 4.
A, Similarity scores of cell clusters from *hGFAP-cre::Crebbp*$^{Fl/Fl}$*::lsl-MYCN* tumors and from V-SVZ/OB cells in the NESFPLO fate mapping model (Ref. 24). B, UMAP plots showing tussie and cluster distribution and expression of aNSC, TAC and NB marker genes in NESFPLO V-SVZ/OB cells.

Supplementary Figure 5

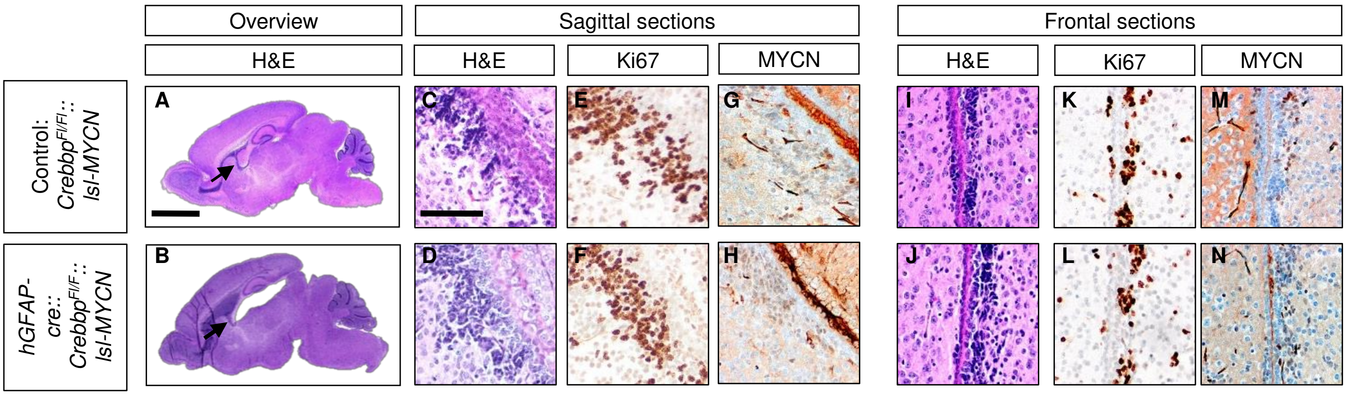

Supplementary Figure 5.

A,B, Histological overview image of brains of p7 mice (*Crebbp*$^{Fl/Fl}$*::lsl-MYCN,* n=3 (A); *hGFAP-cre::Crebbp*$^{Fl/Fl}$*::lsl-MYCN,* n=5 (B)). Arrow marks SVZ. C-H, High power images and IHC of sagittal sections of SVZ of P7 mice. **I-N,** High power images and IHC of frontal sections of SVZ of P7 mice (*Crebbp*$^{Fl/Fl}$*::lsl-MYCN,* n=3 (I,K,M)); *hGFAP-cre::Crebbp*$^{Fl/Fl}$*::lsl-MYCN,* n=3 (J,L,N)). Scale bar corresponds to 2 mm (overview) and 100 µm (high power images).

Supplementary Figure 6

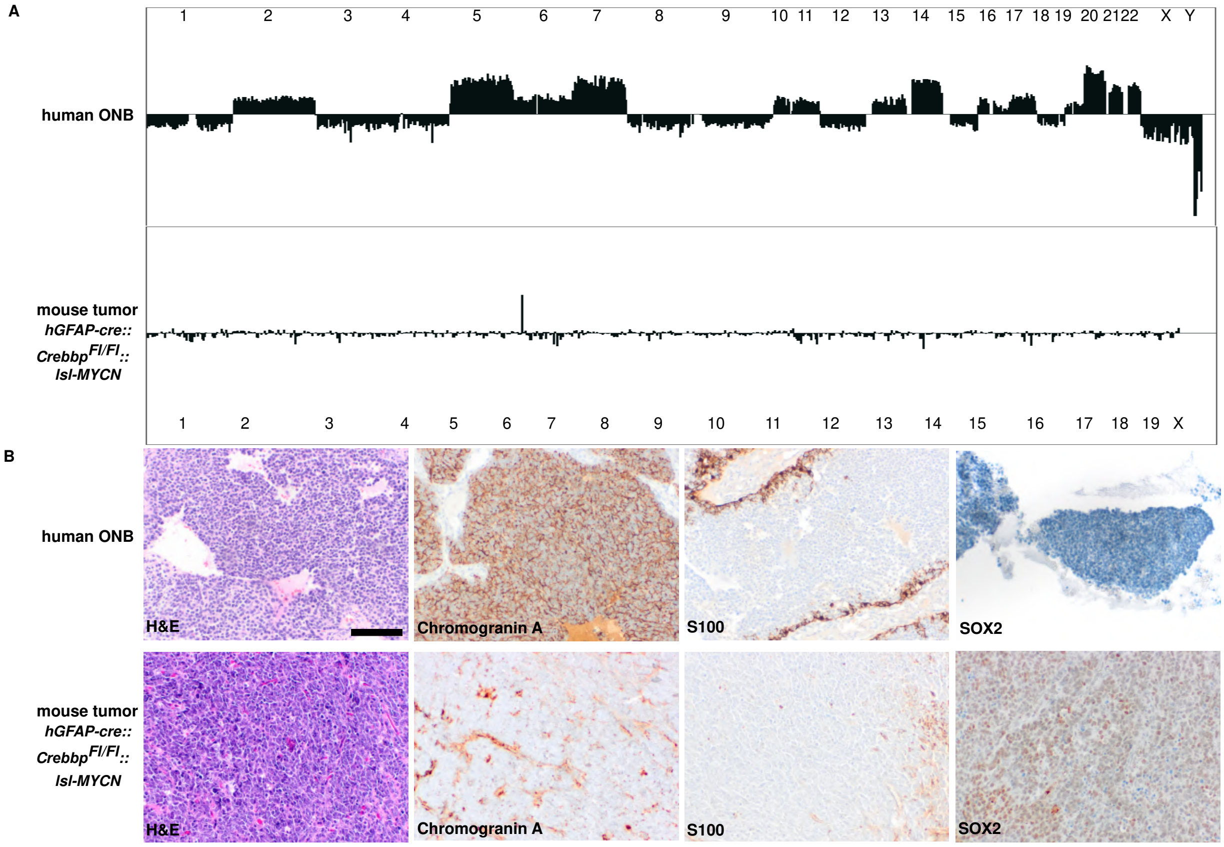

Supplementary Figure 6.
A, Copy number profile comparison of a representative human olfactory neuroblastoma (ONB) and a mouse tumor. The human tumors shows numerours alterations whereas the mouse tumor presents with a relatively flat CNV. (B) Histological comparison of human ONB and mouse tumor. The marker profile of the human tumor is not recapitulated in the mouse tumor. Scale bar corresponds to 100 µm.
